# Supplementary material for: Recurrent episodes of pneumonia are associated with worse lung function in people with HIV
Source: PLoS One. 2026 Apr 13;21(4):e0344756. doi: 10.1371/journal.pone.0344756 (PMC13075695; doi:10.1371/journal.pone.0344756)
Supplement: Attachment — Submitted filename: Response to Reviwers.docx [file pone.0344756.s004.docx]

Sven J. Walderich

University of California, San Francisco

Sven.walderich@ucsf.edu

October 17th, 2025

Re: Manuscript PONE-D-24-50359

Title: Recurrent Episodes of Pneumonia Are Associated with Worse Lung Function in People with HIV

Dear Editorial team and reviewers,

Thank you for the opportunity to revise and resubmit our manuscript to PLOS One. We appreciate the insightful and constructive comments provided by you and the reviewers which have helped us improve the quality of our manuscript.

I removed the financial disclosure statement from the manuscript itself and included a statement that the funders had no involvement in the study design, data collection, analysis, decision to publish or the preparation of the manuscript in my revised cover letter as requested. I added captions for the figures in the supplemental information section of the manuscript. Lastly, I formatted the title as well as author affiliations according to the Journal’s standards.

Please find below our detailed responses to each of the reviewers’ comments.

Please do not hesitate to contact us if further clarification or additional information is needed.

Sincerely,

Sven Walderich

On behalf of all co-authors

**Reviewer 1**

Comment 1: This cohort had a very high rate of current or past smoking (~70+%) and inhalation agent use. While this was 'adjusted' in the models, it is difficult to adjust for such a major common confounder. As such, I recommend conclusion of generalizability to all PWH (first paragraph under discussion and in abstract) should be toned down and caveat of "in this cohort of PWH with a very high prevalence of current/past smoking" should be mentioned. Also if numbers allow, a sensitivity analyses of excluding those with current/past smoking would be of interest.

Response : We agree with the feedback that our cohort had high rates of smoking and inhalational substance use which affects the generalizability of our findings and have revised our abstract as well as the first and final paragraph of our discussion accordingly. In reviewing our data, we did not find a significant difference in the incidence of pneumonia comparing participants who never used inhalational substances including tobacco with those who had a history of prior or current use. We have added these results as Table 1b. Unfortunately, our numbers were not sufficient to conduct a sensitivity analysis among never-smoking participants, though this would be an excellent cohort to investigate in a future study.

Revised Text: “In PWH with a high prevalence of inhalational substance use, recurrent episodes of pneumonia are associated with a commensurate decline in lung function in PWH characterized by airflow obstruction and diffusion limitations.” (page 3, lines 22-24)

“Interestingly, the rate of pneumonia between participants who never used inhalational substances including tobacco and those with a history of prior or current use was similar (Table 1b).” (page 11, lines 4-6)

“In this prospective cohort of PWH with a high prevalence of inhalational substance use, we demonstrate that a single episode of pneumonia is associated with persistent impairment of lung function, including FEV1 %predicted, FVC %predicted, and DLco %predicted. “ (page 14, lines 5-7)

“Another limitation of this study is the high rate of smoking and inhalational substance use among our participants which affects the generalizability of our findings. While the self-reported prevalence of baseline chronic obstructive lung disease was low and the rate of pneumonia was similar with respect to smoking or inhalational substance use history, further studies are needed that include a larger proportion of non-smokers.” (page 17, lines 19-21)

“In conclusion, we found that recurrent episodes of pneumonia in PWH with a high prevalence of inhalational substance use are associated with a commensurate and persistent decline in lung function.” (page 18, lines 10-12)

Comment 2: Manuscript would have been much stronger if measures of QoL/physical functional state was also provided as an outcome (which the authors seem to have collected data on in their cohort). What is the clinical translation of, say, 10% reduction in predicted DLCO or FEV1?

Response: This project had defined objectives focused on pneumonia/recurrent pneumonia/types of pneumonia (predictors) and lung function measurements (outcomes). Our group has previously shown that a reduced DLCO (predictor) is associated with increased respiratory symptom burden (modified Medical Research Council dyspnea scale, COPD Assessment Test score and St. George’s Respiratory Questionnaire) as well as increased mortality(outcomes) (PMIDs: 37463173 and 40504822), which are currently referenced in our discussion section. In addition, other studies have demonstrated an association between respiratory symptoms and a decline in spirometry (PMID 12762353), which is why we focused this study on objective lung function parameters. Your question is an important one, but also one that would require a different design (possibly longitudinal), as well as different predictors (change/reduction in lung function), and different outcomes (QoL). As we continue to gather more participants with more longitudinal values we plan to address the impact on quality of life measures in future projects.

Revised Text: “The finding that recurrent pneumonia can result in the development of an isolated reduction in the diffusing capacity without concomitant airflow obstruction carries important clinical implications as prior studies have shown that iso↓DLco is associated with increased morbidity and mortality (27,28,35,36)”. (page 16, lines 2-5)

Comment 3: Given high prevalence of smoking, how was COPD handled? The lung function decline will depend heavily on background COPD severity and optimal management. At least provide data on COPD prevalence and adjust for.

Response: We appreciate this feedback and have included data on baseline prevalence of self-reported COPD (n=9, 4%) as well as other pulmonary comorbidities in Table 1. It is true that the decline in lung function is affected by COPD. However, given the low baseline prevalence of COPD in our cohort we did not adjust for this comorbidity in our models but rather included smoking as a covariable. We did complete an analysis among participants without airflow obstruction and observed a similar association between recurrent pneumonias and a decline of DLCO (Table 4).

Revised Text: “A minority of patients had self-reported underlying pulmonary diseases including asthma (20%), chronic obstructive pulmonary disease (4%) and interstitial lung disease (1%).” (page 10, lines 19-20)

Comment 4: Is there anyway to adjust/exclude viral pneumonias? Wonder if those would have a different effect?

Response: This is an especially interesting point considering the recent COVID pandemic. Unfortunately, the number of viral pneumonias (13, or 8%) was insufficient to determine a differential impact on long-term lung function compared to other etiologies. We have added the prevalence of viral pneumonia to Table 1.

Comment 5: Any data on vaccinations (viral or pneumococcal) that can also affect the results and should be easy to extract from EMR?

Response: We thank the reviewer for this important point. We did not have immunization data for our study participants. We have added some additional text about the potential role of immunization on our findings in the discussion.

Revised Text: “While pneumonia incidence in PWH has improved in the contemporary ART era, it remains a significant source of morbidity and mortality for this population. More broadly, pneumonia prevention through the routine use of vaccination remains an important public health priority. Pneumococcal vaccination is recommended for all adults with HIV due to its efficacy in pneumonia prevention. Unfortunately, we did not have access to vaccination records in this analysis and could not assess the role of vaccination on pneumonia prevalence, and, indirectly, lung function decline. However, it is conceivable that adequate vaccination against pneumococcus, SARS-CoV-2 or influenza may be even more critical in PWH than in people without HIV for the preservation of lung function. “ (page 17, lines 4-12)

Comment 6: Provide IQR after the sentence: "The median time between the most recent episode of pneumonia and pulmonary function testing was 148 days..."

Response: We have included the IQR and added the data point to Table 1a. After reviewing our data, we discovered that the previously reported median time between the most recent episode of pneumonia and pulmonary function test (148 days) was inaccurate; this has now been corrected.

Revised Text: “The median time between the most recent episode of pneumonia and pulmonary function testing was 436 days (IQR 172, 1485 days).” (page 11, lines 6-8)

**Reviewer 2**

Comment 1: The discussion repeats many results rather than interpreting them. Please focus more on mechanisms, public health/clinical implications, and comparison with prior studies.

Response: We have revised the discussion by limiting the reiteration of results to the first paragraph and have added additional interpretation in the subsequent paragraphs. We have also added a paragraph on the public health and clinical implications.

Revised Text: “While pneumonia incidence in PWH has improved in the contemporary ART era, it remains a significant source of morbidity and mortality for this population. More broadly, pneumonia prevention through the routine use of vaccination remains an important public health priority. Pneumococcal vaccination is recommended for all adults with HIV due to its efficacy in pneumonia prevention. Unfortunately, we did not have access to vaccination records in this analysis and could not assess the role of vaccination on pneumonia prevalence, and, indirectly, lung function decline. However, it is conceivable that adequate vaccination against pneumococcus, SARS-CoV-2 or influenza may be even more critical in PWH than in people without HIV for the preservation of lung function. “ (page 17, lines 4-12)

Comment 2: Limitations are acknowledged, but some key ones are missing (e.g., self-reported measures, cross-sectional design limiting causal inference, potential selection bias).

Response: We have added additional limitations to the discussion section.

Revised Text: “Another limitation of this study is the high rate of smoking and inhalational substance use among our participants which affects the generalizability of our findings. While the self-reported prevalence of baseline chronic obstructive lung disease was low and the rate of pneumonia was similar with respect to smoking or inhalational substance use history, further studies are needed that include a larger proportion of non-smokers. Given the cross-sectional nature of this study, we were unable to establish causality and many of our measures relied on self-report. Lastly, selection bias may have occurred if participants with respiratory symptoms related to abnormal lung function were more likely to attend outpatient visits and be enrolled in our study, leading to an overestimation of the magnitude of our findings” (page 17, lines 19-25 and page 18, lines 1-3)

Comment 3: The conclusion should be more concise and aligned with the findings. Avoid making causal claims if the study design is observational.

Response: We appreciate this comment. We have revised our wording in the conclusion to avoid any claims of causality.

Revised Text: “In conclusion, we found that recurrent episodes of pneumonia in PWH with a high prevalence of inhalational substance use are associated with a commensurate and persistent decline in lung function.” (page 18, lines 10-12)
